# Computational Modeling of DNA 3D Structures: From Dynamics and Mechanics to Folding

**DOI:** 10.3390/molecules28124833

**Published:** 2023-06-17

**Authors:** Zi-Chun Mu, Ya-Lan Tan, Jie Liu, Ben-Gong Zhang, Ya-Zhou Shi

**Affiliations:** 1 Research Center of Nonlinear Science, School of Mathematical & Physical Sciences, Wuhan Textile University, Wuhan 430073, China; 2 School of Computer Science and Artificial Intelligence, Wuhan Textile University, Wuhan 430073, China

**Keywords:** DNA 3D structures, computational modeling, molecular dynamics simulations, coarse-grained models, structure fragment assembly

## Abstract

DNA carries the genetic information required for the synthesis of RNA and proteins and plays an important role in many processes of biological development. Understanding the three-dimensional (3D) structures and dynamics of DNA is crucial for understanding their biological functions and guiding the development of novel materials. In this review, we discuss the recent advancements in computer methods for studying DNA 3D structures. This includes molecular dynamics simulations to analyze DNA dynamics, flexibility, and ion binding. We also explore various coarse-grained models used for DNA structure prediction or folding, along with fragment assembly methods for constructing DNA 3D structures. Furthermore, we also discuss the advantages and disadvantages of these methods and highlight their differences.

## 1. Introduction

**Figure 1:**
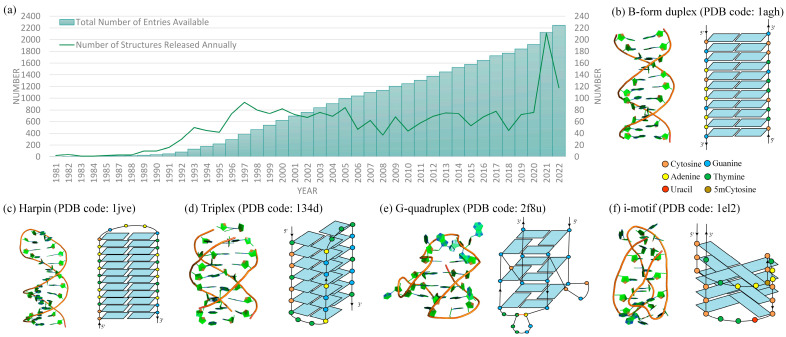
(**a**) DNA-only structures released in Protein Data Bank (PDB) (http://www.rcsb.org/, accessed on 1 October 2022) per year. Bars: total number of entries available. Line: number of structures released annually. (**b**–**f**) Three-dimensional (**left**) and secondary (**right**) structures for (**b**) B-form dsDNA and typical non-B form DNAs: (**c**) DNA hairpin; (**d**) triplex; (**e**) G-quadruplex; (**f**) i-motif. The 3D structures are shown with PyMol (http://www.pymol.org, accessed on 1 October 2022).

Since the genetic information encoded by DNA forms the basis of life [1,2], the exploration of its structure and stability is a thriving field. For instance, the number of known functional DNA structures in the Protein Data Bank (PDB) continues to increase each year (see Figure 1a). In terms of stability, most DNA exhibits a right-handed double helix structure (B-form, see Figure 1b). This structure follows the Watson–Crick–Franklin law of A-T and G-C base-pairing, serving as a carrier for storing and transmitting genetic information in living organisms [3]. However, recent research has indicated that roughly 13% of human genes can adopt non-right-handed double helix structures (non-B-form) [4,5,6,7,8,9,10], such as hairpins [4], Z-DNA [5], triplexes [7], G-quadruplexes [8,9,10], and i-motifs [4,5] (see Figure 1c–f). These structures have been observed to play significant roles in various cellular processes, including gene expression regulation and cancer development [4,5,6]. For example, DNA triplexes are generally involved in mutagenesis, genetic instability, and DNA repair or recombination, and mutations in helicases that act on G-quadruplex structures could lead to DNA damage or replication errors [7,8,9]. In addition, DNA nanostructures and devices (e.g., interlocks, walkers, tweezers, motors, shuttles, logic circuits, and origami) have immense potential for applications in various fields such as biosensing, food safety, and cancer therapy [11,12,13].

The function of DNA often depends on its 3D structure [3,4,7,8,9]. For example, the dynamically interchangeable G-quadruplex structures in HIV-1 can be stabilized by ligand binding, resulting in decreased viral production [14]. Therefore, understanding the 3D structures and properties of DNA (e.g., dynamics, thermodynamics, and mechanics) is useful in understanding its biological functions and designing DNA nanomaterials [2,3,4,5,6,14]. However, the flexibility and polymorphism of DNA present challenges for current experimental techniques, such as cryo-electron microscopy, X-ray crystallography, NMR spectroscopy, and other single-molecule techniques (e.g., light/magnetic tweezers and atomic force microscopy) [15,16,17,18]. These experimental methods face difficulties in elucidating the underlying aspects of DNA folding, hybridization, and stability. Since these methods are often time-consuming and costly, the number of known DNA structures in the database is still very limited (see Figure 1a).

The field of computer simulation is advancing quickly, providing more precise insights into essential aspects of DNA biophysics compared to traditional experimental approaches [19,20,21,22]. Molecular dynamics (MD) simulations, for instance, can generally reproduce the behavior of molecules in a computer, providing detailed structural and dynamical insights that enhancing our comprehension of relevant experimental data. In recent years, MD simulations using classical force fields such as AMBER [23,24] and CHARMM [25] have provided highly detailed and flexible descriptions of DNA dynamics, including structural transformations, stability of non-canonical conformations, salt ion cohesion effects, twist-stretch coupling of stress, flexibility under methylation modifications, and interactions with other macromolecules. It is always fascinating to obtain microscopic insights into DNA dynamics through MD simulations. However, the innumerable degrees of freedom, interconnected in complex ways, can make it practically impossible to detect DNA dynamics on biologically relevant time scales and length scales using currently available computer hardware [26].

In contrast to all-atom models, continuous DNA models such as the worm-like chain (WLC) model effectively describe the mechanical behavior of double-stranded DNA (dsDNA) on larger length scales. This model considers the double helix as an elastic rod with torsional and bending stiffness (i.e., the predefined angle between neighbor beads and persistence length of the chain) [27,28,29]. Similarly, the nearest neighbor model can predict the secondary structure and melting profiles (such as free energy and melting temperature) of single-stranded DNA (ssDNA) and dsDNA. This model assumes that the free energy of DNA is the sum of the free energy of each base stack, which has been determined through thermodynamic experiments [30,31]. However, these basic models are unable to provide insights into the three-dimensional (3D) structures of DNA.

Meanwhile, coarse-grained (CG) models, which combine highly correlated atoms in the DNA nucleotide into a few interacting sites, can play a crucial role in describing complex biological macromolecular systems (e.g., DNA–protein complexes and DNA nanostructures) at larger length/time scales [32,33]. Compared to all-atom models with large numbers of particles, CG models have a small number of degrees of freedom due to the reduced resolution. CG models are generally effective in studying DNA 3D structures, dynamics, flexibility, and interactions with other biological macromolecules (such as RNA and protein) [34,35,36]. However, all-atom MD simulations for DNA generally require known 3D structures as input. Although many models have been developed for RNA 3D structure prediction [37,38,39,40], there are few methods that can be directly used to predict DNA 3D structures, especially from the sequence. Recently, Xiao et al. provided a fragment assembly method (3dDNA) to automatically predict 3D structures for small DNAs (<100 nt) with very high precision [41,42,43,44]. Since the method depends on limited templates and known secondary structures of the DNA, more DNA 3D structure prediction models, especially ab initio ones, are still needed.

In this work, we provide a comprehensive review of computer modeling techniques used for studying DNA 3D structures. Our goal is to provide an in-depth understanding of the current state-of-the-art research, as well as to discuss the challenges and future developments in the field of DNA 3D structure modeling. First, we reviewed the powerful and versatile all-atom MD simulations, including progress and limitations in capturing DNA dynamics, flexibility, and ionic interactions. Then, we highlighted representative DNA CG models that exhibit excellent performance in DNA folding or simulations for large DNAs beyond the capabilities of all-atom MD simulations. Finally, since MD simulations and several CG models generally require known 3D structures, we provided a brief overview of current structure assembly methods that can construct 3D structures of DNA from their sequences or secondary structures.

## 2. Molecular Dynamics Simulations for DNAs

**Figure 2:**
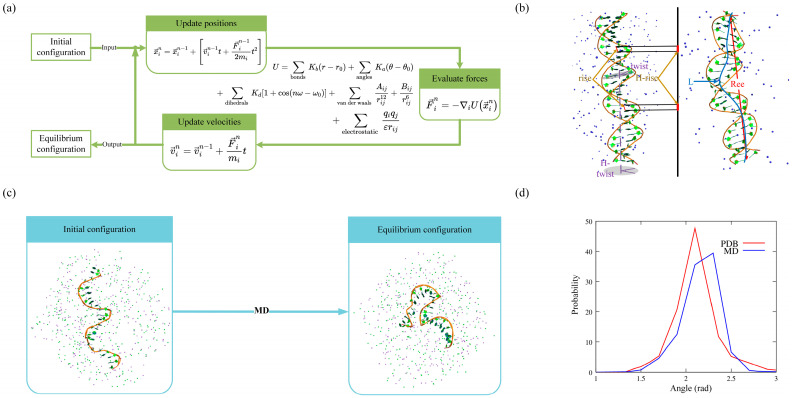
(**a**) Schematic diagram of MD simulations. (**b**) Diagram of calculations on the flexibility of dsDNA in MD simulations. Left: initial conformation. Right: equilibrium conformation. (**c**) MD simulations for short ssDNA in ion solutions. (**d**) Distributions of the angle between contiguous P-C4′-P atoms from experimental structures (red) and MD simulated conformations (blue). The 3D structures are shown with PyMol (http://www.pymol.org).

MD simulations of DNA systems are typically performed by calculating the force on each atom as a function of their positions using all-atomic force fields (such as AMBER, CHARMM, GROMOS, and OPLS) (Figure 2a). These force fields are parameterized using experiments or quantum chemistry calculations of small systems [23,24,25,45,46,47]. CHARMM36 [25] and AMBER ff99bsc1 [47], which have been validated and improved through multiple revisions, are commonly used for DNA simulations. Although these force fields have limitations, such as AMBER potentially overestimating base stacking effects and CHARMM weakening base pairing [48,49], they have been successfully employed to simulate DNA systems, providing atomistic resolution and establishing quantitative relationships between structure and conformational energy [50,51].

### 2.1. Structural Dynamics

MD simulations have been effective in accurately probing the atomic motions and structural dynamics of DNAs [52,53,54,55,56,57,58,59], enabling us to understand the DNA functions. To address the question of how long an MD simulation of a B-DNA helix needs to be to sample the dominant structural and dynamical features, Galindo-Murillo et al. presented an extensive analysis using multiple μs-length MD simulations of a dsDNA (d(GCACGAACGAACGAACGC)) with Amber 14 and a ff99SB parmbsc0 or CHARMM C36 force field on multiple computer architectures (including Anton, CPU, and GPU). The results showed that despite the underlying differences in hardware, the simulations performed on different architectures exhibited minimal structural variation with respect to one another. These MD simulations, including the longest one at ~44 μs, also suggested that the structure and dynamics of the DNA helix, excluding the terminal base pairs, reach near-full convergence on the ~1–5 μs timescale. This indicates that the current force field is reasonably robust. However, the convergence of the terminal base pair opening events occurs on time scales significantly longer than 10 μs and cannot be fully captured through ensembles of shorter and independent MD simulations [60,61]. In a separate study, Yang et al. performed umbrella MD simulations of A-T sequence-rich B-DNA using the Amber force field and reproduced the experimental conformational transition path from Watson–Crick to Hoogsteen base pairs observed in NMR relaxation dispersion spectroscopy [62]. This indicates that MD simulations have the power to describe large-scale structural dynamics at short timescales using an advanced-sampling approach [63].

In addition, MD simulations can also provide detailed insight into DNA structure dynamics. For example, Chakraborty et al. employed the AMBER12 package and Joung/Cheatham ion parameters to explore the transition between B- and Z-dsDNAs. Their study found that the free energy landscape exhibits two distinct funnels, leading to the B-DNA and Z-DNA conformations. This suggests that the reversal of chirality is caused by the stretched DNA structure or mutual competition at the B–Z junction [64].

### 2.2. Structural Flexibility

In recent years, MD simulations have been widely used to study the flexibility of DNAs, as DNA structural flexibility is closely associated with many biological processes involving the storage or encoding of genetic information [65,66]. Although many results from single-molecule experiments can be well-described by the commonly accepted WLC models [27,28,29], atomistic MD simulations are extensively used to obtain microscopic descriptions of DNA flexibility, such as the width and depth of the major/minor grooves and the distances/twist angles between neighbor base pairs [67,68,69]. For example, to explain the experimental results that short DNAs consisting of tens of base pairs (bps) may have seemingly higher flexibility than those of kilobase pairs, Wu et al. performed MD simulations for short dsDNAs with a finite-length of 5–50 bps using the Amber parmbsc0 force field. Their microscopic analyses (the calculation of stretching and bending at the base-pair level) revealed that the apparent high flexibility of short dsDNAs arises from significantly strong bending and stretching flexibilities at each helix end, consisting of ∼6 bps [70]. In addition to the length-dependent flexibility of DNA, Marin-Gonzalez et al. performed over 1μs-long constant-force MD simulations of 18 bp-long dsDNAs (CGCG(NN)_5_CGCG, with NN as the AA, AC, AG, AT, CG, and GG). They found that the DNA crookedness (a sequence-dependent deformation of DNA that consists of periodic bends in the chain of base pair centers) and its associated flexibility can regulate DNA mechanical properties at short length scales. This unveiled a one-to-one relation between DNA structure and dynamics [71]. To understand the distinct differences in the flexibility of dsRNA and dsDNA helices, Liebl et al. performed unrestrained/restrained MD simulations for a 16 bp dsDNA or dsRNA using the AMBER12 package with the parmbsc0 force field. Their detailed analysis of helical deformations, coupled with twist, indicated that the interplay of helical rise, base pair inclination, and displacement from the helix axis during twist changes is responsible for the different twist–stretch correlations [72]. Coincidentally, Marin-Gonzalez et al. investigated the difference between dsDNA and dsRNA (16 bp) using microsecond-long MD simulations under constant stretching forces within the range of 1–20 pN. They showed that the opposite twist–stretch coupling of both molecules is due to the markedly different evolution of inter-strand distance with the stretching force, which is directly correlated with the slide base-pair parameter and sugar pucker angle [73]. Recently, Bao et al. also conducted extensive MD simulations for larger dsDNA and dsRNA (40 bp) without applying stretch force, using the AMBER ff99bsc0 force field. Their work provides a more quantitative understanding of the relative flexibility of dsRNA and dsDNA in terms of both stretching and twist–stretch coupling. They noted that the striking difference in twist–stretch coupling between dsRNA and dsDNA is attributed to the apparently stronger base-pair inclination in dsRNA compared to dsDNA (Figure 2b) [74].

In addition, MD simulations can be used to reproduce the effect of base modifications or base-pair mutations on DNA flexibility [75,76]. For example, Aksimentiev et al. combined MD simulations (using the NAMD program with a CHARMM36 force field) with a single-molecule cyclization assay to study how different cytosine modifications influence the physical properties of dsDNA (70 bp). They elucidated the microscopic mechanisms behind the changes in DNA flexibility induced by cytosine modifications: these modifications can promote or dampen structural fluctuations through the competing effects of base polarity and steric hindrance [77]. Given that the appearance of mismatched base pairs (MMs) can result in the development of inherited genetic diseases, cancer, and aging, Rossetti et al. presented the first comprehensive study on the structure of MM-containing DNA duplexes (12 MMs, including A·A, A·C, A·G, C·A, C·C, C·T, G·A, G·G, G·T, T·C, T·G, and T·T, placed in the center of 13 bp duplexes, e.g., d(CCATACXATACGG)). They employed MD simulations (Gromacs v.4.5.5 program with parmbsc0 force field) and NMR spectroscopy and found that the presence of mismatches produced significant local structural alterations due to the flexible MMs (especially in the case of purine transversions). These alterations could be propagated far from the mismatch site, influencing the global structures of DNA [78]. On the other hand, Bouchal et al. also employed MD simulations (Amber 16 package with parmbsc1 force field) to calculate the thermodynamic stabilities of MMs in similar dsDNAs (e.g., d(GGTTAAXTTAACC) with anti/anti, anti/syn, and syn/anti MM combinations) as a function of two geometry parameters of the base pair (opening and shear). However, their detailed analysis showed that there was no clear dissection between the canonical and mismatched base pairs [79]. This discrepancy suggests that MD simulations may be less credible in capturing the local sequence effects on DNA flexibility [74] due to the empirical force field.

### 2.3. DNA–Ion Interaction

Since DNA is an anionic polyelectrolyte, the solvent environment plays a significant role in DNA structures [80,81,82,83,84]. Pasi et al. performed microsecond MD simulations for 39 dsDNAs (with a length of 18 bp and different sequences) under physiological salt conditions using the parmbsc0 force field with Dang parameters for the ions. They provided a comprehensive state-of-the-art perspective on sequence-dependent potassium ion populations. For example, they observed that potassium ions within the grooves are more likely to accumulate around electronegative base sites rather than the anionic phosphate groups [85]. Considering the experimental results showing that high-valent cation can lead to the opposite effect on the elasticities of DNA and RNA duplexes, Fu et al. used MD simulations for 20 bp dsDNA and dsRNA in trivalent ion solutions (i.e., CoHex^3+^). They found that these results were caused by different binding modes of the cations on dsDNA and dsRNA [86]. More recently, Cruz-Leon et al. also combined high-resolution MT experiments with MD simulations (parmbsc1 force field on 33 bp dsDNA) to show that increasing ion concentration leads to a decrease in helical radius and crookedness, an increase in sugar pucker, and ultimately an increase in a twist. This is due to the increased screening of electrostatic repulsion between phosphate groups [87].

Furthermore, MD can provide an atomistic understanding of how DNA–ion interactions vary with different metal ions (Figure 2b,c). For example, Long et al. performed MD simulations to sample the structures of a 23 bp DNA duplex in various ion solutions (such as Mg^2+^, Ca^2+^, Sr^2+^, or Ba^2+^). They demonstrated that these ions exhibit a preference for binding to the phosphate backbone rather than the major groove [88]. To investigate the competitive binding of divalent and monovalent ions to dsDNA, Xi et al. performed all-atom MD simulations for a 24 bp dsDNA in mixed Mg^2+^/Na^+^ solutions using the Amber parmbsc0 force field with Joung/Cheatham ion model for Na^+^/Cl^−^ and the Aqvist ion model for Mg^2+^. Their comprehensive analysis suggested that the global binding of Mg^2+^ over Na^+^ to nucleic acids is primarily dependent on the surface charge density and Mg^2+^/Na^+^ concentrations [89].

### 2.4. Limitations

In the last 40 years, MD simulations have made significant progress in providing atomistic insights into DNA structures, including dynamics, flexibility, and ion binding. Although recent efforts combining experiments and simulations show promise for improving the accuracy of nucleic acid force fields, MD simulations are not always effective, particularly for ssDNAs [90,91]. Recently, we performed MD simulations for unstructured ssDNA (with a random sequence: 5′-CTGCCACGCCATGCCTGTGACGA-3′ at 1 M [Na^+^]) and tried to extract the bonded parameters from the equilibrium conformations. However, we found that the distributions of several angles in MD conformations deviated from those observed in PDB structures. For example, the P-C4′-P angle showed a deviation of ~11° from its optimal value, as shown in Figure 2d. In addition, the ion parameters, which are optimized based on a set of experimental solution properties such as solvation-free energies, radial distribution functions, water exchange rates, and activity coefficient derivatives, could be limited in their transferability to quantitatively describe biomolecular systems [92,93]. Thus, further investigations of diverse DNA structures (e.g., ssDNA, pseudoknots, G-quadruplexes, i-motifs, and DNA complexes) in ion solutions are needed to further assess the quality of these force fields [90,91,94,95].

Furthermore, MD simulations in equilibrium are not always adequate to sufficiently explore the structural space needed for accurate property estimation [96,97]. In MD simulations, the initial conformation is usually established based on an experimentally known structure. If the molecule acquires another stable conformation that is separated by a high free energy barrier, the system’s acquisition of this alternative conformation within a realistic computational cost becomes challenging due to the barrier [91]. Finally, sampling remains an issue in some nucleic acid simulations, thus requiring the extension of simulation time scales and exploration of efficient enhanced sampling methods (e.g., temperature replica exchange, Hamiltonian and multi-dimensional replica exchange, metadynamics, and umbrella sampling). These efforts are important for future advancements [98,99,100].

## 3. Coarse-Grained (CG) Modeling for DNAs

Due to the computational limitations of MD simulations, CG models are often utilized to study DNA structure folding, such as hybridization and melting. These models reduce the complexity of atomistic simulation systems by averaging nonessential degrees of freedom [32,33]. There are currently two primary approaches to CG DNA modeling: top-down, which involves parameter fitting to experimental data, and bottom-up, which involves analyzing parameters from all-atom MD simulations or quantum chemistry calculations [34]. Based on their design purpose and capability, existing DNA CG models fall into three main categories: 3D structure dynamics, properties/folding, and prediction [32,33,34] (see Table 1).

### 3.1. CG Models for DNA Structure Dynamics

Since MD simulations are limited by computational cost at different length scales and time scales (typically ranging from nanoseconds to milliseconds), CG MD simulations can be a suitable for accelerating the study of large DNA structures.

**Figure 3:**
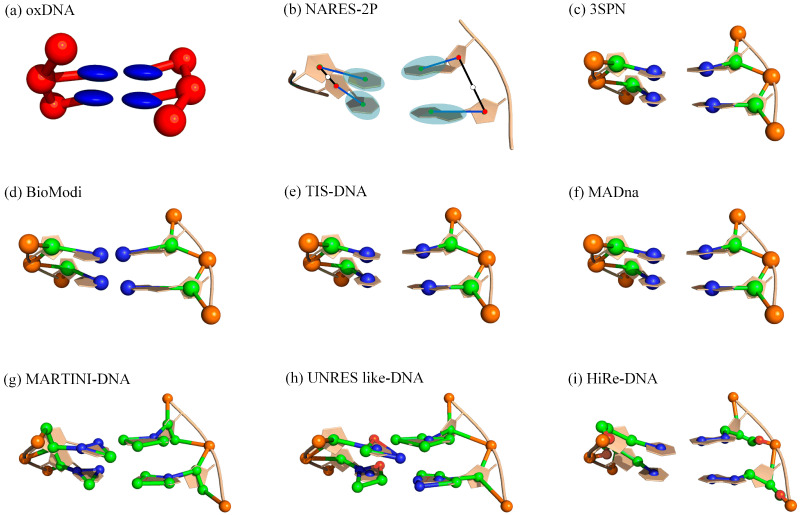
Representations of several DNA CG models. (**a**) oxDNA: two beads [104,105,106,107]. (**b**) NARES-2P: the united sugar bases (B’s) and the united phosphate groups (P’s) serve as interaction sites [108,109]. (**c**) 3SPN: three beads [110,111,112,113]. (**d**) BioModi: three beads [114]. (**e**) TIS-DNA: three beads [115]. (**f**) MADna: three beads [116]. (**g**) MARTINI-DNA: six/seven beads [102]. (**h**) UNRES-like DNA: six/seven/eight beads [117]. (**i**) HiRE-DNA: six/seven beads [118]. All 3D structures (ball-stick: CG; cartoon: all-atom) are shown with PyMol (http://www.pymol.org).

For this purpose, Marrink et al. proposed an explicit solvent-based DNA CG model that is compatible with the Martini force fields, suited for MD simulations of biomolecular systems [101,102,103]. In the Martini DNA model, each nucleotide is mapped to six or seven CG beads, with one bead for the phosphate group, two for the sugar ring, and three (four) for the pyrimidines (purines) (see Figure 3g). Similar to the Martini protein force field [101], the model incorporates conventional bonded (bond length, angle, and dihedral angle) and nonbonded interactions (Lennard–Jones potential and Coulombic energy) for DNA. In addition, a new interaction was added to model directional hydrogen bonds in DNA. The force field was parameterized by combining top-down information from experiments with bottom-up information derived from reference all-atom MD simulations. For the bonded parameters, all-atom and CG MD simulations were performed on 10 ssDNAs with different sequences, respectively, and the parameters were adjusted to match the conformations available in the Martini force field to the conformational space of the reference all-atom model as closely as possible. The nonbonded parameters were derived from partitioning the nucleobases between polar and nonpolar solvents, as well as the base–base potential of mean force calculations. The model was validated by reproducing the radius of gyration of ssDNA, as well as the double helical structures and persistence length of dsDNA, as observed in atomistic simulations under high ion concentrations. It is important to note that, for dsDNA, an elastic network (which involved predefining the pairing bases and adding interactions between them) was used in the Martini model to preserve the secondary structure. Although the Martini DNA model cannot be used to study DNA hybridization, melting, and hairpin formation due to its inability to model directional hydrogen bonds, its speed and compatibility pave the way for large-scale modeling of complex biomolecular systems involving DNA, such as DNA–protein interactions [102].

Recently, another sequence-dependent CG model (MADna) was proposed by Assenza and Pérez for simulating dsDNA [116]. In the MADna model, each nucleotide is represented by three effective particles located at the geometric centers of the phosphate group, sugar, and base (see Figure 3f). In the model, the sequence-dependent bonded interactions including bond, angle, and dihedral potentials are used to connect beads within the same strand as well as to provide inter-strand links (e.g., between beads in preassigned pairing nucleotides). These interactions are tuned to reproduce the results of atomistic simulations of dsDNAs with various sequences. In addition, the model includes an excluded-volume interaction implemented through the repulsive component of a Lennard–Jones interaction, and the salt-induced electrostatic was modeled via a Debye–Hückel (DH) interaction (between P beads with a charge of −0.6). By combining with LAMMPS, the MADna can capture the sequence-dependence of conformational and elastic properties of dsDNA, including main helix parameters, groove geometry, the diameter of the double helix, and spontaneous curvature quantified by bending metrics, with an accuracy comparable to atomistic simulations. Furthermore, the model can reproduce structural elastic features observed in experiments, such as the stretching and torsion moduli, negative twist–stretch coupling, twist–bend coupling, persistence length, and helical pitch. However, due to the double-stranded structure imposed by the bonded interactions in MADna, it cannot account for breaking events such as the formation of kinks or local melting.

**Table 1:** Existing DNA structure modeling models/methods.

Models	Representation	Type ^a^	Application ^b^	Available ^c^
Hall et al. [119]	1 bead	Gō-like	Duplex/Triplex *T_m_*	/
Aksimentiev et al. [77]	2 beads	ab initio	*R_g_*, *L_p_*, force-extension	/
oxDNA [104,105,106,107]	2 beads	Gō-like	*T_m_*, *L_p_*, force-extension, hybridization, dynamics, DNA–ion interaction, and nanotechnology	https://oxdna.org (accessed on 1 October 2022)
NARES-2P [108,109]	2 beads	ab initio	3D structure prediction, *T_m_*, dynamics	/
Mittal et al. [120]	2 beads	Gō-like	*T_m_*, Particle interactions	/
MaDNA [116]	3 beads	MD	dsDNA structure/elastic properties, *L_p_*	https://github.com/saassenza/MADna (accessed on 1 October 2022)
3SPN [110,111,112,113]	3 beads	Gō-like	*T_m_*, *L_p_*, structure properties, dynamics, hybridization, DNA–ion interaction, nanotechnology	https://github.com/depablogroup (accessed on 1 October 2022)
TIS [115]	3 beads	Gō-like	*R_g_*, *L_p_*, *T_m_*, force extension,	/
Plotkin et al. [121]	3 beads	ab initio	*L_p_*, DNA twist, and stacking	/
Shi et al. [122]	3 beads	ab initio	3D structure prediction, salt effect, *T_m_*, *L_p_*	https://github.com/RNA-folding-lab/DNAfold (accessed on 1 October 2022)
BioModi [114]	3 beads	Gō-like	Hybridization and self-assembly kinetics, salt-dependent *L_p_*	/
Dorfman et al. [123,124,125]	3 beads	ab initio	*T_m_*, dynamics, structure properties, triplex forming	/
Nordenskiöld et al. [126]	5 beads	MD	dsDNA *L_p_*, *L_T_*	/
SIRAH [127]	6 beads	MD	dsDNA *T_m_*, transitions, and dynamics	/
“sugar” CG [128]	6 beads	MD	dsDNA transitions, DNA–ion interaction	/
MARTINI [102]	6/7 beads	MD	*R_g_*, *L_p_*, 3D structure, DNA–ion interaction, DNA–protein complexes	http://cgmartini.nl/ (accessed on 1 October 2022)
HiRe-DNA [118]	6/7 beads	ab initio	dsDNA 3D structure, *T_m_*	/
UNRES like-DNA [117]	6/7/8 beads	ab initio	dsDNA 3D structure, structure properties, and hybridization	/
3dDNA [44]	all-atom	structure assembly	3D structure prediction for DNAs with single, double, and multi-chains	http://biophy.hust.edu.cn/new/3dRNA (accessed on 1 October 2022)
Saiz et al. [129]	all-atom	structure assembly	ssDNA 3D structure prediction	/
Rahim et al. [130]	all-atom	structure assembly	ssDNA 3D structure prediction	/

^a^ ab initio: modeling DNA structure from sequence only; Gō-like: predefined secondary structure or base-pairing network is needed; MD: 3D structure is needed; structure assembly: constructing DNA 3D structures based on the secondary structure. ^b^ indicates what the models can be used for. *T_m_*: melting temperature; *R_g_*: radius of gyration; *L_p_*: persistence length; *L_T_*: torsion persistence length. ^c^ indicates the open source code or web server of each model/method, and ‘/’ indicates that the model is unavailable.

### 3.2. CG Models for DNA Structure Folding

Since the above CG models developed for MD simulations require known DNA 3D structures as input, it is difficult to use them to study DNA folding processes such as hybridization, melting, and hairpin formation.

Generally, the Gō-type model is very effective in studying the folding of macromolecules (protein, RNA, and DNA) [104,105,106,107,110,111,112,113,115,131,132,133,134,135]. It achieves this by only considering the interactions that occur at the given native contact sites. The oxDNA is one outstanding representative of this model, which can capture the structural, thermodynamic, and mechanical properties of DNA [104,105,106,107]. In this model, DNA is represented as a string of rigid nucleotides with interaction sites for backbone, stacking, and hydrogen bonding interactions (see Figure 3a). The pairwise potential comprises eight interactions (see Table 2), including connectivity between neighboring backbones, the favorable stacking interactions between adjacent bases, coaxial stacking, and electrostatic repulsive interactions. The model was parameterized using a heuristic top-down approach, which involved reproducing well-known properties of DNA (such as the helical structure of dsDNA) and experimental results (such as melting temperatures of ds/ssDNAs). Combined with the virtual moving Monte Carlo algorithm or LAMMPS simulation software, this model has provided key insights into many different processes relevant to DNA nanotechnology and biophysics. It has also provided direct agreement with experimentally measured properties across a range of systems, including duplex hybridization, hairpin formation, DNA overstretching, thermodynamics, and structural properties of ss/dsDNAs.

The 3SPN model is another three-site per nucleotide model, with one site each for the phosphate, sugar, and base, thereby rendering the investigation of DNA up to a few microns in length computationally tractable [110,111,112,113] (see Figure 3c). In 3SPN, the potential energy of a DNA system comprises seven distinct contributions (Table 2), including typical bonded potentials (intramolecular bonds, bond angles, and dihedral angles) and pairwise nonbonded interactions (e.g., intra-strand base stacking, inter-strand cross-stacking, base pairing, excluded volume contributions, and electrostatic potential). The model is parametrized using thermal denaturation experimental data at a fixed salt concentration. Through replica exchange MD simulations, the 3SPN has been found to effectively reproduce many sequence/salt-dependent structural and mechanical properties of ds/ssDNAs, such as local flexibilities, minor groove width profiles, persistence lengths, melting temperatures, and hybridization rate.

Similar to 3SPN, a new three-interaction site model (TIS) has also been developed to provide a robust description of the sequence-dependent mechanical and thermodynamic properties of ss/ds DNAs [115]. The TIS model includes sequence-dependent stacking, hydrogen bonding, and electrostatic interactions, as well as bond-stretching and bond angle potentials (Table 2). The force constants for the stretching and bending potentials were guided by a Boltzmann inversion procedure using a large representative set of DNA PDB structures, and the parameters in the stacking interactions were calculated using a learning procedure, ensuring faithful reproduction of experimentally measured melting temperatures (i.e., a top-down approach). The model can accurately predict the salt-dependent persistence lengths of ss/dsDNA and melting temperatures of DNA hairpins, which represent a significant improvement over most of the current CG models.

### 3.3. Ab Initio CG Models

The CG models introduced in the previous section typically utilize a Gō-type potential, which imposes penalties on deviations from a reference structure, to constrain the range of conformations explored by a CG model of DNA. However, this approach also has the potential to restrict the ability of the model to accurately predict structures based solely on sequence information.

In contrast to the Gō-like models mentioned above, Plotkin et al. introduced an alternative CG model for DNA [121] that does not use any structure-based potential. In this model, phosphate and sugar groups are represented by one CG spherical residue each, while bases are represented by rigid-body ellipsoids to model their stereochemistry. The total potential includes eight purely physicochemical interactions (Table 2). In addition to the usual local bonded interactions, the model includes electrostatic repulsion interaction between phosphates, van der Walls interactions between any two beads, and base–base hydrogen bonding. These effective interactions were parameterized through all-atom simulations. For example, local potentials along the backbone were obtained from the statistics on conformations obtained from all-atom simulations, and base–base/backbone interactions were obtained from the best fit between van der Waals interactions in an all-atom model and an anisotropic potential between effective ellipsoids. By employing the LAMMPS package, the model generated stable double-stranded helices with both major and minor grooves for dsDNA and predicted the persistence lengths for ss/dsDNA that were comparable to experimental values. Furthermore, the model examined the degree of stacking and twist as functions of temperature, salt concentration, and sequence for ss/dsDNA.

UNRES-like DNA is a physics-based middle-resolution CG model [117]. In this model, the sugar (S) is represented by a neutral bead, the phosphate (P) is represented by a negatively charged bead, and each base (B) is reduced to a set of rigid bipolar beads (e.g., 4 for T and 5 for A) (see Figure 3h). The total potential energy is a summation of ten interaction potentials (Table 2). The parameters of bonded interactions were derived to reproduce the behavior of model systems in the all-atom representation. Nonbonded interactions were approximated using Lennard–Jones, excluded volume, and electrostatic interactions of charges and dipoles. The model was parameterized in a bottom-up fashion with only small adjustments to obtain the correct balance of key interactions. Using an efficient *R*-RATTLE rigid-body integration algorithm, the model successfully folded three short dsDNAs from separated complementary strands, despite underestimation of persistence lengths of ss/dsDNA.

HiRE-RNA is another high-resolution CG model designed for both RNA and DNA. In this model, each nucleotide is represented by six or seven beads: one for the phosphate (P), four for the sugar, and one/two for the pyrimidine/purine bases [118,136] (see Figure 3i). The force field of this model is expressed as a sum of local bonded, nonbonded, electrostatic, and hydrogen-bond terms (Table 2). Notably, the hydrogen bond interactions in the model consist of three terms: a two-body interaction (distance and angle), a three-body term (to avoid multiple hydrogen bonds of just one base), and a four-body term (representing stacking between two base pairs). The equilibrium geometrical parameters were initially derived from known structures and subsequently refined through the analysis of long MD simulations for a 15 nt Poly(A) molecule. By using replica exchange molecular dynamics (REMD), the model can accurately be used to predict the correct double helix structure from a completely random configuration and allows for the study of dissociation curves as well as the sequence effect on the melting curves of the duplexes.

On the other hand, NARES-2P is a physics-based CG DNA model with only two interaction sites: one for phosphate (P) and one for the base (B) (see Figure 3b) [108,109]. Similar to in UNRES [117], the effective energy function of the NARES-2P model originates from the PMF of a polynucleotide in water. The energy includes van der Waals or electrostatic interactions between any two beads, virtual bonded interactions, and sugar–base–rotamer energy terms (Table 2). Additionally, a restraint energy was also introduced to maintain selected geometric parameters (e.g., site–site distances) within the desired range. These potential energy terms were parameterized using Boltzmann inversion and fitting the PMF calculated by the all-atomic potential energy surface. The NARES-2P model was built into the UNRES/MD platform, which enables canonical and replica-exchange simulations of nucleic acids to be carried out. Through a global-optimization conformational space annealing algorithm, the model can not only find the native fold for simple DNA duplexes but also reproduce the thermodynamics of folding, although the calculated melting temperatures are generally higher than the experimental values.

**Figure 4:**
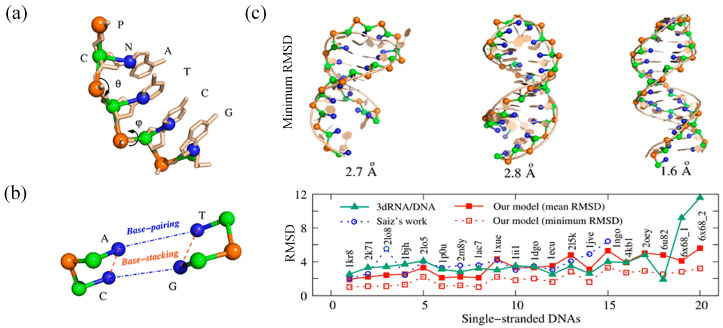
(**a**) CG representation of a DNA fragment in our model superimposed on the all-atom representation. (**b**) Schematic representation of base-pairing and base-stacking interaction. (**c**) 3D structures of ssDNAs predicted by our model compared with existing models.

Recently, we have also presented a new CG model to fold DNA 3D structures based only on the sequence. In this model, each nucleotide is simplified to three beads corresponding to the phosphate (with a negative charge), sugar, and base (see Figure 4a). The total energy of the system is composed of eight potentials, similar to the RNA CG model previously developed by our team [122,137,138,139,140,141]. The parameters for the bonded potentials, including bond length, bond angle, and bond dihedral, were derived from the Boltzmann inversion of the corresponding atomistic distribution functions obtained through statistical analysis of the experimental structures from the PDB. The excluded volume interaction between the CG beads is modeled by a purely repulsive Lennard–Jones potential. The orientation-dependent base-pairing interaction for the possible canonical Watson–Crick base pairs does not require any predefined structural information (see Figure 4b). The sequence-dependent base-stacking and coaxial-stacking (see Figure 4b) were parameterized using well-established experimental DNA thermodynamic parameters [30,142], and a conformational entropy change was included in the Monte Carlo simulation. It is important to note that the electrostatic interactions between the phosphate beads were also taken into account using the DH approximation, in combination with the counterion condensation theory and tightly-bound ion model [143,144], to predict DNA structures in monovalent/divalent ion solutions.

Using the effective Monte Carlo simulated annealing algorithm, the model successfully folded 20 dsDNAs (≤52 nt) and 20 ssDNAs (≤74 nt) into the corresponding native-like structures based on their sequences, with an overall mean RMSD of 3.4 Å from the experimental structures (Figure 4c).

**Figure 5:**
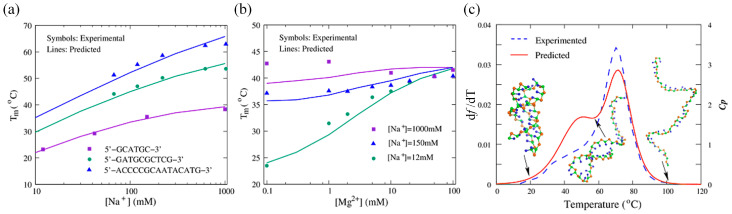
(**a**) Melting temperatures as functions of [Na^+^] for three dsDNAs with different sequences. (**b**) Melting temperatures as functions of [Mg^2+^] for the dsDNA at different [Na^+^]s. (**c**) Comparisons between predictions and experiments for a DNA pseudoknot at 0.1 M [Na^+^].

Furthermore, the model quantitatively predicted the thermodynamic stability of 27 dsDNAs (including bulge loops and internal loops) and 24 ssDNAs (including a double hairpin and a pseudoknot), with a mean deviation of predicted melting temperatures from the corresponding experimental data of only ~2.0 °C (Figure 5). For example, the predicted two transformation temperatures (~48.8 °C and ~72.0 °C) for a DNA pseudoknot at 0.1 M [Na^+^] closely match the experimental data (~52.6 °C and ~70.7 °C), as shown in Figure 5c. Furthermore, the model also reproduced the stability of ssDNAs/dsDNAs under extensive monovalent or mixed monovalent/divalent ion conditions, with the predicted melting temperatures consistent with the available experiments (Figure 5).

Despite recent advancements, the present ab initio models have limitations in predicting large DNAs with complex structures, indicating the need for further improvement in the energy function and sampling methods [145,146,147,148].

### 3.4. Discussion and Comparison of These CG Models

As shown in Table 1 and Figure 3, the reduced degree of freedom is different for various CG models. For instance, oxDNA uses two beads, TIS uses three beads, and HiRE uses six or seven beads [104,115,118]. Generally, elaborate models can capture more detailed interactions, but they may be limited in structure modeling for large DNAs. For example, although the two, three, and four-body hydrogen bond interactions can be defined in the HiRE model, it is only applicable in small DNAs (<100 nt) [118]. Conversely, the oxDNA model can be used to simulate DNA nanostructures (>1000 nt) [107]. Predefined secondary structure information (i.e., Gō-like) is very important for CG models to simulate large DNAs [104,105,106,107,110,111,112,113].

Furthermore, these models were designed for different purposes, as outlined in Table 1. Some are suitable for CG MD simulations to capture DNA structure dynamics at large time and length scales (e.g., Martini and MADna) [102,116,126,127,128]. These models can reproduce details of DNA structures (e.g., helix parameters and groove geometry) and structural elastic features (e.g., persistence length and twist–stretch coupling) in most cases [102,116]. However, they generally require native/near-native 3D structures as inputs. Some other CG models were developed to simulate DNA folding, such as oxDNA, 3SPN, and TIS, which can be used to predict the thermodynamic or kinetic properties (such as melting temperatures or folding rates) of DNA [104,112,115]. In order to ensure that the DNA can fold into the correct final structure, additional secondary structure constraints are usually necessary in these models. Moreover, some ab initio CG models (such as HiRE, NARES-2P, and our model) have also been proposed to simulate 3D structure folding for DNA based only on its sequence [77,108,117,118,121,122,123]. Notably, these models can be used to predict 3D DNA structures, as well as their corresponding thermodynamic stability. However, they are only applicable to small DNAs (<100 nt).

## 4. DNA Structure Assembly Method for 3D Structure Construction

Since all-atom MD simulations for DNAs generally require known 3D structures as input, and DNA nanostructures are generally assembled by simple fragments (e.g., double helices), it is crucial to quickly build DNA 3D structures from sequences, especially for large DNAs. In this section, we will review several DNA structure assembly methods based on DNA secondary structures.

**Figure 6:**
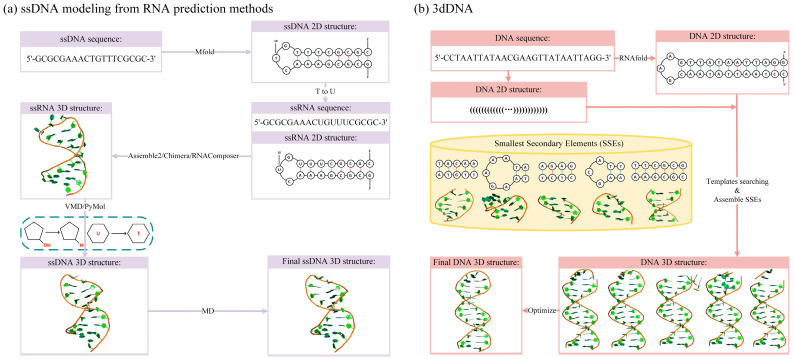
Workflow of DNA structure assembly method. (**a**) Workflow of two indirect 3D structure prediction methods for ssDNA with the aid of the RNA structure prediction methods [129,130]. (**b**) Workflow of 3dDNA for DNA 3D structure prediction [44].

Due to considerable progress in RNA 3D structure prediction [37,38,39,40], two indirect ssDNA 3D structure prediction methods have been proposed with the aid of RNA models [129,130]. For example, in the pipeline presented by Saiz et al., a secondary structure was first predicted using Mfold [31] based on the given sequence. Subsequently, a corresponding 3D RNA structure was constructed using RNA structure prediction methods (such as Assemble and RNAComposer). The 3D RNA structure was then converted into a DNA structure by replacing the nucleotide U with T, and the resulting 3D structures were refined through energy minimization, as shown in Figure 6a. Although these methods were only tested on several small ssDNA hairpins (7–27 nt) and their accuracy was not very high (the RMSDs between predicted and experimental structures were larger than 4.0 Å), they offered a new framework for investigating related ssDNA nanotechnology.

Recently, Xiao et al. proposed a direct template-based method, 3dDNA, which is an extension of their previous 3dRNA. This method aims to construct DNA 3D structures by assembling 3D templates of the smallest secondary elements (SSEs) [44], as illustrated in Figure 6b. First, DNA is decomposed into SSEs based on the given secondary structural information. Second, the corresponding 3D template for each SSE can be found in the well-defined DNA fragment library. Subsequently, the selected template of each SSE is assembled with its parent SSE by superposing them using the Kabsch algorithm, with reference to the two common base pairs. The resulting assembly models are further refined by minimizing them using the AMBER force field to repair the chain connectivity of the assembled structures. To evaluate the performance of 3dDNA, it was was benchmarked on three test sets with different numbers of chains. The results showed that 3dDNA can predict DNA 3D structures with a mean RMSD of approximately 2.36 Å for structures with one or two chains, and fewer than 4 Å for structures with three or more chains. These results indicate a significant improvement compared to the indirect methods [44,129,130].

Since these fragment assembly methods heavily rely on the known secondary structure, which can be challenging to determine or predict accurately, especially for large complex DNAs, achieving accurate predictions of DNA 3D structures still seems to be a long-term challenge.

## 5. Discussion

The rapid advancement of MD simulations and DNA modeling has led to extensive insights into DNA structures at both macroscopic and microscopic scales [32,33,34,50,51,90]. However, the increasing utilization of DNA-based bioengineering and nanotechnology, as well as the discovery of non-B DNA structures with unique biological functions, has further intensified the requirement for DNA modeling. Here, we reviewed the recent advancements in DNA structure dynamics and folding, including MD simulations, CG modeling, and fragment assembly. Our purpose was to enhance DNA structure-based applications and further promote the development of DNA modeling.

In addition to the methods reviewed above, many computational models specially designed for DNA nanostructure construction or simulation have also been developed (e.g., MrDNA, DAEDALUS, and Adenita) [149,150,151]. Due to space limitations, we cannot delve into all of them in detail. Furthermore, the field of biology has seen significant advancements in recent years due to the application of machine learning techniques [152,153,154,155,156]. For example, 3D structure prediction methods such as AlphaFold2 [157] and RoseTTAFold [158] have gained popularity due to their ability to accurately predict protein structures. These deep learning methods could also improve the accuracy of DNA simulations by capturing more complex interactions between atoms whenever possible. However, since deep learning models require large datasets for training, the limited number of known DNA structures challenges the application of these methods in DNA modeling. With the development of advanced hardware, highly accurate force fields, large amounts of experimental data, and refined computer modeling techniques, DNA modeling has the potential to not only explain a large number of experimental results [69,86,87], but also to serve as a guiding tool for new and exciting discoveries [159,160].

## Figures and Tables

**Table 2:** Usual potentials explicitly used in typical DNA CG models ^a^.

	Potential	$U_{b}$	$U_{a}$	$U_{d}$	$U_{exc}$	$U_{bp}$	$U_{bs}$	$U_{cs}$	$U_{el}$	$U_{pp}$	$U_{ps}$	$U_{pb}$	$U_{ss}$	$U_{sb}$	$U_{bb}$
Model															
oxDNA		√			√	√	√	√	√	√					
3SPN		√	√	√	√	√	√	√	√						
TIS		√	√		√	√	√		√						
Plotkin et al.		√	√	√		√				√		√		√	√
UNRES-like DNA		√	√	√						√	√	√	√	√	√
HiRE-DNA		√	√	√		√				√					
NARES-2P		√	√							√		√			
Shi et al.		√	√	√	√	√	√	√	√						

^a^ indicates the main potentials used in typical DNA CG models, and √ indicates that the potential is explicitly included in the model. $U_{b}$, $U_{a}$, and $U_{d}$ are potentials of bond length, angle, and dihedral for neighbor CG beads, respectively. $U_{exc}$: excluded volume interaction; $U_{bp}$: base pairing or hydrogen bonding interactions; $U_{bs}$: base stacking interactions; $U_{cs}$: coaxial stacking interactions; $U_{el}$: electrostatic repulsive interactions; $U_{pp}$, $U_{ps}$, and $U_{pb}$ are interactions between phosphate and-phosphate/sugar/base; $U_{ss}$ and $U_{sb}$ are interactions between sugar and sugar/base, respectively; and $U_{bb}$: base–base interactions.

## Data Availability

There is no new data were created.
